# Hydrophilic gold nanospheres: influence of alendronate, memantine, and tobramycin on morphostructural features

**DOI:** 10.1186/s13065-022-00891-1

**Published:** 2022-11-22

**Authors:** Dina M. Eissa, Mokhtar M. Mabrouk, El Zeiny M. Ebeid, Mohamed A. Abdel Hamid

**Affiliations:** 1 Ministry of Health and Population, Menoufia, Egypt; 2 Pharmaceutical Analytical Chemistry Department, Faculty of Pharmacy, Tanta University, Elgeish Street, Tanta, 31111 Egypt; 3 Chemistry Department, Faculty of Science, Tanta University, Elgeish Street, Tanta, 31111 Egypt

**Keywords:** Gold nanospheres, Alendronate, Memantine, Tobramycin, Stability, Biocompatibility, Drug targeting

## Abstract

Turkevich gold nanospheres are the original nanospheres that have been modified over time. Its combination with targeting medications such as alendronate, memantine, and tobramycin will provide additional benefits in targeting specific areas in the bone, brain, and microorganisms, respectively. Hence, The reactivity and stability of nanospheres with various drug concentrations (milli-,micro-, and nano-levels) have been studied. With alendronate, the absorbance spectra of nanospheres at $\uplambda _{max}$ 520 nm were always stable and no redshifts occurred. In contrast, the spectra with memantine and tobramycin were stable at the nano-level and redshifts occurred at the milli- and micro-levels. HRTEM and DLS revealed that the core diameter was relatively stable in all cases, whereas the hydrodynamic diameter and zeta potential varied with varying drug concentrations. Increasing concentration increased hydrodynamic diameter slightly with memantine (from 64.99 to 98.41 nm), dramatically with tobramycin (from 135.3 to 332.16 nm), and almost negligibly with alendronate (from 52.08 to 58.94 nm ). Zeta Potential, conversely, is reduced as concentration increases. Memantine had the greatest reduction in negativity, followed by tobramycin, but alendronate had a slight increase in negativity. Benefits from this research would be in targeted drug delivery, where stability and reactivity of gold nanospheres are critical.

## Introduction

Gold chemistry is a rapidly growing era as gold is a unique noble metal, having distinguished properties among other metals such as thermal and chemical stability, electrical conductivity, mechanical softness, and the highest electronegativity among all metals [1]. Gold metal nanostructures differ in color and properties from bulk metal [2]. In addition to the fundamental characteristics of nanomaterials, gold metal nanostructures exhibit a variety of optical, physical, and chemical characteristics including excellent localized surface plasmon resonance (LSPR, strong oscillation of the electron cloud of metal nanospheres induced by incident light), surface-enhanced Raman scattering, photoluminescence, resistance, bioinert nature, photothermal effect ...etc. These properties make them interesting for biomedical [3, 4], biosensing, bioimaging, genetic engineering [5], photothermal therapy [6], theranostic [7–10], and drug delivery applications [11–13].

Structure symmetric isotropic gold nanospheres (AuNSs)’ advantages including large surface-to-volume (S/V) ratio, biocompatibility, and ease of surface modification, make them more vulnerable to surface functionalization. The use of functionalized AuNSs in pharmaceuticals has been continuing to enhance efficacy, raise systemic bioavailability, and mitigate side effects [11].

These nanospheres are very well known from ancient times [14]. Nowadays, AuNSs are widely used nano-carriers in targeted drug delivery systems (TDDSs) [15–17]. TDDSs are relatively new techniques for delivering drugs directly to specific sites (organ, receptor, etc...). The main goal of these techniques is to reduce side effects while increasing the therapeutic value through enhancing targetability. Loading gold nanospheres by drug molecules is one of the techniques used to achieve targeted delivery. Nanospheres drug delivery depends on certain physicochemical merits such as particle size, morphology, hydrophilicity, hydrodynamic diameter, stability, surface charge, and reactivity [18–20].

Alendronate sodium (Alen) is a bisphosphonate that has been used to treat a variety of bone conditions, including osteoporosis, Paget’s disease, and malignant hypercalcemia [21]. Due to the strong hydrophilicity and intense polarity of bisphosphonate medications, Alen cannot easily pass the cell membrane. Its oral absorption effectiveness is really poor. On the other hand, Alen is harmful to soft tissues and can trigger pancreatitis and mouth ulcers. Due to its gastrointestinal irritant, higher doses of Alen provide a higher risk of adverse effects. Therefore, it is crucial to find new ways to deliver Alen to the target site in effective and low doses [22]. One of these ways is utilizing the nano-features of gold nanospheres to improve the bioavailability and distribution of Alen.

Memantine is frequently employed to treat memory loss, disorientation, and issues with thinking and reasoning which are cognitive signs of Alzheimer’s disease. Memantine’s function as an uncompetitive (open-channel) NMDA receptor antagonist, which prevents glutamate from acting on this receptor, is most likely the mechanism by which the medication has its pharmacological impact. Memantine prefers to interact with cation channels controlled by the N-methyl-D-aspartate (NMDA) receptor. Despite these antagonistic effects, memantine has not been shown to stop or slow the neurodegeneration seen in people with Alzheimer’s disease [23, 24]. Nano-delivery of memantine using AuNSs may improve its pharmacological effect.

Bactericidal aminoglycoside tobramycin [25] is active against a variety of Gram-negative and certain Gram-positive bacteria. It is a polycationic antibiotic that rapidly binds to bacterial membranes at physiological pH. (a process known as “ionic binding”). This includes binding to teichoic acid and phospholipids found in the cell membrane of Gram-positive bacteria as well as phospholipids and lipopolysaccharide found in the outer membrane of Gram-negative bacteria. Due to this interaction, divalent cations are displaced, the membrane becomes more permeable, and aminoglycoside access results [11]. The proton-motive force is needed for additional aminoglycoside entrance into the cytoplasm, which enables the aminoglycoside to reach its main intracellular target, the bacterial 30S ribosome. More aminoglycoside can enter the cell as a result of the disruption of the cell membrane caused by the mistranslated proteins that are created as a result of aminoglycoside attaching to the ribosome. As a result, tobramycin and other aminoglycosides have bactericidal effects that are both immediate through membrane disruption and delayed through decreased protein synthesis [26, 27].

All previous medications are freely soluble in water and no need for other solvents which may affect or disrupt the stability of hydrophilic gold nanospheres.

The stability and mechanism of gold nanospheres’ aggregation with targeting medications without interfering with external factors, does not have enough attention, as a result, light has been shed on the way the nanospheres may interact and aggregate with such medications that have specific targets, like alendronate (Alen), memantine (Mema), and tobramycin (Tobr). These medications target bone marrow, brain receptors, and various Gram-negative Gram-positive bacteria, respectively. Targeting these sites is challenging and important in the therapeutic management of various diseases such as Osteoporosis [28], Alzheimer’s [29], and Cystic fibrosis [30].

Several studies investigated the stability and reactivity of gold nanospheres with targeted medications using external factors such as buffer, temp. ...etc. However, few studies focused on the stability and reactivity of AuNSs with medications without any external factors affecting either drugs or nanoparticles. In this study, gold nanospheres’ morphostructural features, stability, and reactivity to various concentrations (milli-, micro-, and nano-) of alendronate, memantine, and tobramycin, without other external factors, have been investigated.

## Experimental

### **Apparatus**

All spectra analysis were done on a Jasco UV-Vis spectrophotometer model-(V-530) (Japan), a Jasco Fourier Transform Infrared (FT/IR) Spectrometer model-4100 (Japan) and a JASCO spectrofluorometer model-FP-6300 (Japan). Edwards Modulyo Freeze Dryer for lyophilization of prepared samples before FTIR measurments. Gold nanospheres core diameter was measured by High Resolution Transmission Electron Microscopy (HRTEM) model-(JEOL JEM-2100), while nanospheres hydrodynamic diameter and surface charge (zeta potential) analyzed by Malvern Zetasizer (Nano-ZS90). PH measurements measured by WTW inoLab$\circledR$ Multi 9620 IDS Multiparameter Benchtop Meter. Automatic Water Still. Electronic Balance. Micro Quartz Cuvette (1 ml). 250 ml single-neck flask with flat base. Distillation and reflux condenser. Thermo Fisher Scientific^TM^ hotplate and stirrer.

### **Materials**

Hydrogen tetrachloroaurate(III) hydrate ($HAuCl_{4}.3H_{2}O$ , M.W: 339.79 $g.mole^{-1}$, $\backsim$ 52% Au basis, anhydrous basis) purchased from sigma aldrich, Egypt. Trisodium citrate dihydrate ($Na_{3}C_{6}H_{5}O_{7}.2H_{2}O$, M.W.: 294.10 $g.mole^{-1}$, $> 99.0$ %) purchased from Gateway Co. Double Distilled Water freshly obtained from Automatic Water Still, for all preparations, and rinsing thoroughly all glasswares. Alendronate sodium ($C_{4}H_{12}NNaO_{7}P_{2}$, M.W: 271.08 $g mole^{-1}$), memantine ($C_{12}H_{21}N$, M.W: 179.30 $gmole^{-1}$) and Tobramycin base ($C_{18}H_{37}N_{5}O_{9}$, M.W: 467.45 $gmole^{-1}$) purchased from sigma aldrich, Egypt. Aqua Regia (1:3 mixture of concentrated concentrated nitric acid ($HNO_{3}$) to concentrated hydrochloric acid (HCl)) used continuously to wash out all glasswares before and after each procedure.

### **Methods**

#### **Gold nanospheres synthesis**

Synthesis of gold nanospheres was done by the following clear steps as mentioned in previous literature [31]:- All glassware was rinsed and soaked in aqua-regia for 1 h, cleaned thoroughly with double distilled water, and dried in an oven. A 25 mM Chloroauric acid $(HAuCl_{4})$ (M.W.: 339.79 $gmole^{-1}$) solution was prepared by dissolving 85 mg $(HAuCl_{4})$ in double distilled water in a 10 ml volumetric flask. At the same time, in a 100 ml volumetric flask, 38.8 mM trisodium citrate dihydrate $( Na_{3}C_{6}H_{5}O_{7} \cdot 2H_{2}O)$, (M.W.: 294.10 $gmole^{-1}$) solution was prepared by dissolving 1.1411 g in double distilled water and volume completed to 100 ml by double distilled water. After that, a 4 ml of previously prepared solution of 25 mM $HAuCl_{4}$ was added into a clean 250 ml flat bottom necked flask containing 96 ml distilled water and a magnetic bar. That flask was connected to a condenser to reflux at 1100 rpm and heat up to boiling at 100 $^{\circ }$ for 45 min with continuous stirring. During the boiling of the solution, the stirring process turned off and 10 ml of 38.8 mM trisodium citrate dihydrate was added instantly at once and the pale yellow color disappeared. The stirring is turned on again, and the color started to change from black to deep violet to red-purple. lastly, the solution refluxed for another 20 min till the color is fixed, and the hotplate was turned off, leaving the solution to cool with continuous stirring. The prepared solution has been centrifuged at 13500 rpm for 30 min, and the precipitate only was re-suspended again with double distilled water. The colloidal solution was stored in tightly closed amber glass surrounded by aluminum foil in a refrigerator at 4 $^{\circ }$. The colloidal solution showed stability for several months, except if not stored very well.

#### **Standard solutions**

Each medication was precisely weighed at 10 mg, transferred to a 10 ml volumetric flask, and volume completed with double distilled water to create a 1 $mgml^{-1}$ stock standard solution. Next, serial dilutions were performed, where 1 ml of each drug’s stock solution was added to a 100 ml volumetric flask to make 10 $ugml^{-1}$ working standard solutions, and 1 ml of the 10 $ugml^{-1}$ working solutions was added to additional 100 ml volumetric flasks to create 100 $ngml^{-1}$ working standard solutions. Stock and working standard solutions were always freshly prepared.

#### **Reaction procedures **

Various concentrations of alendronate (Alen), memantine (Mema), and tobramycin (Tobr) were prepared in situ (in a micro cuvette) directly before spectrophotometric measurement using 1 $mg ml^{-1}$, 10 $ug ml^{-1}$ and $100 ng ml^{-1}$ working standard solutions. A final volume settled to be $1000 \, ul$. Different aliquots of working solutions were taken and volumes were completed to the final volume by diluted AuNSs (1:5). The concentrations were prepared in the milli-, micro-, and nano-ranges for each drug. The nano-ranges of Mema and Tobr were (33.46–334.63 nM), and (4.28–128.34 nM), while the micro-ranges were (0–2.23 uM) and( 0–2.139 uM), respectively. Alen concentration was in the milli-ranges (0.184–2.582 mM). Within twenty minutes, numerous measurements of each concentration were made against a blank solution with proper shaking in between each measurement. Linearity was known by plotting $(\textit{A}_{0}-\textit{A}_{n})/\textit{A}_{0}$ against the wide range of concentrations of Alen, Mema, and Tobr, where ($\textit{A}_{0}$) is the absorbance of AuNSs, ($\textit{A}_{n}$) is the absorbance of AuNSs loaded by (*n*) concentrations of the selected medications. All spectra were measured through a wavelength range of (400–600 nm) and maximum absorption wavelength at $\uplambda _{max}$ 520 nm. The reactions of AuNSs with Alen, Mema, and Tobr have been optimized with the study of various factors such as multiple drug concentrations, reaction time, and dilutional effect. Concentrations in the milli-, micro-, and nano-scales have been studied. The reaction of AuNSs with each medication has been studied within twenty minute. Also, the final volume of AuNSs has been studied. The pH of the prepared solutions has been measured and recorded in triplicate using pH meter.

#### **Gold nanospheres characterization**

The color change, morphology, structure, chemical composition, stability, and reactivity of gold nanospheres have been examined using the following characterization techniques.

- *UV-Vis spectroscopy* The absorbance of AuNSs in the presence and absence of the three medications at milli-, micro-, and nano-concentrations has been measured by UV-Vis spectrophotometer at $\uplambda _{max}$ 520 nm. The absorbance measurement on the UV-Vis spectrometer was done through a wavelength range of (200–800 nm). A $1.5 \, ml$ of freshly prepared AuNSs were transferred to a Quartz cuvette for direct measurement against a blank of double distilled water.
- *High-resolution transmission electron microscopy (HRTEM)* Morphology and core diameter of AuNSs were measured using HRTEM in the presence and absence of the three medications. Micro- and nano-concentrations of 10 $ug ml^{-1}$ and 100 $ng ml^{-1}$ working standard solutions have been utilized. That is to determine whether the color change is due to a change in the nanosphere core diameter, or if the color change is simply due to only nanosphere aggregation by other mechanisms.
- *Malvern Zetasizer* Malvern Zetasizer was used to analyze the hydrodynamic diameter and zeta potential of AuNSs in the presence and absence of the three medications at the micro- and nano-concentrations. The modification in surface charge and hydrodynamic diameter of the nanocrystals caused by the addition of various concentrations of the three medications should be investigated to predict how these particles may aggregate.
- *Fourier-transform infrared spectroscopy (FTIR)* FT/IR Spectrometer was used to analyze and investigate the chemical and functional group composition of the prepared solutions of gold nanospheres. All samples have been lyophilized before FT/IR measures using Edwards Modulyo Freeze Dryer. All spectra have been recorded in the mid-IR range of 400–4000 $cm^{-1}$ before and after the addition of alendronate, memantine, and tobramycin. Micro- and nano-concentrations of 10 $ug ml^{-1}$ and 100 $ng ml^{-1}$ working standard solutions have been used. Investigating their interactions can be done by comparing the gold nanospheres’ FT/IR record when used alone and when combined with the chosen medications in the nano- and micro-concentrations.

All characterization measurements were done at room temperature (25 $^{\circ }$ ).The final solution in each procedure was always a 4:1 mixture of gold nanospheres ’ solution to medication solutions.

## Results and discussion

### Gold nanospheres’ concentration and extinction coefficient

**Fig. 1:**
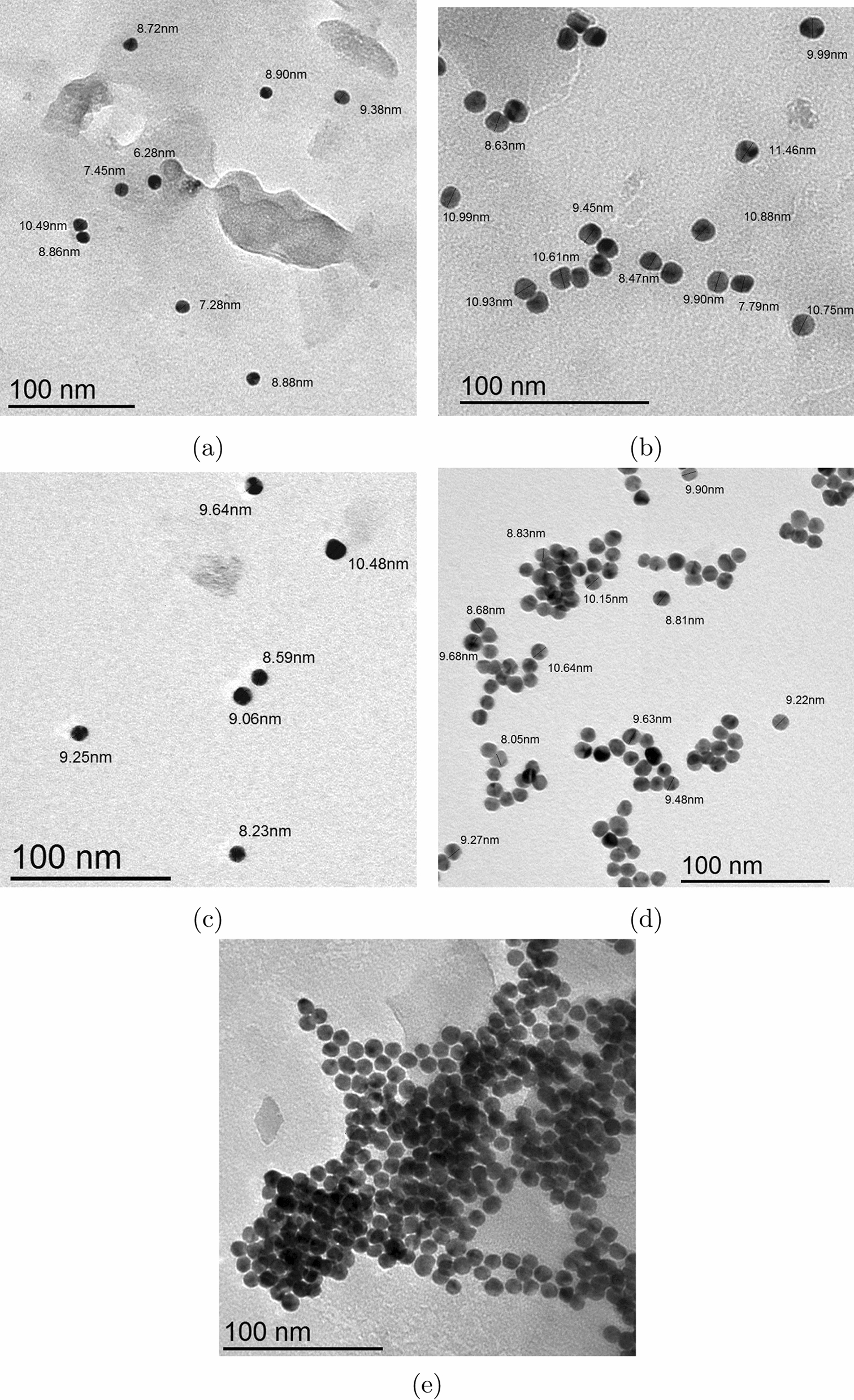
High resolution transmission electron microscope (HRTEM) images of gold nanospheres, alone (**a**), with tobramycin nano-concentration (**b**), with alendronate micro-concentration (**c**), with memantine micro-concentration (**d**), with tobramycin micro-concentration (**e**)

**Table 1:** Mean molar concentrations $^{a}$ and extinction coefficients ($\epsilon$)$^{b}$ of gold nanosphere solutions

Sample	AuNSs’ molar conc. ± SD (M)	$\epsilon \pm SD (M^{-1} cm^{-1})$	Drug conc.
AuNSs	$3.68 * 10^{-8}$		—
AuNSs with Alen	$(3.97 \pm 0.29)10^{-8}$	$(3.02 \pm 0.39) 10^{4}$	3.69 mM
AuNSs with Mema	$(3.67 \pm 0.36 )10^{-8}$	$(2.22 \pm 0.029) 10^{6}$	55.77 uM
AuNSs with Tobr	$(3.22 \pm 0.084)10^{-8}$	$(5.35 \pm 0.047) 10^{6}$	213.90 nM
AuNSs with Tobr	$(3.33 \pm 0.37)10^{-8}$	——-$^{c}$	21.39 uM

$^{a}$ molar concentrations calculated from Eq. (2)

**Fig. 2:**
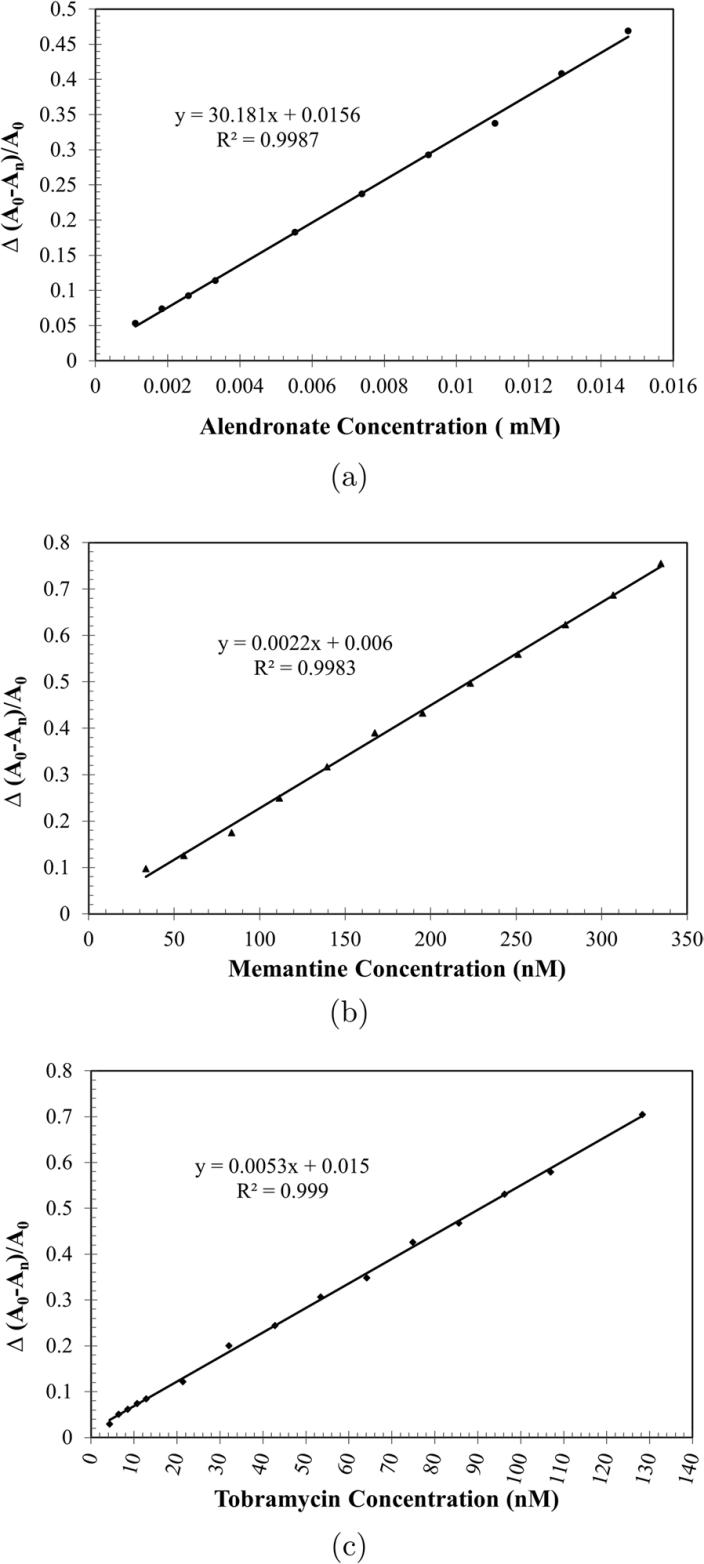
Linearity curve and correlation coefficient of gold nanospheres loaded by: **a** alendronate milli-concentration within range of (0.11–1.48 E−02 mM). **b** Memantine nano-concentration within range of (33.46–334.63 nM). **c** Tobramycin nano-concentration within range of (4.28–128.34 nM)

$^{b}$ extinction coefficients calculated from the slope of regression lines in Fig. (2)

$^{c}$ extinction coefficient not calculated due to aggregation of AuNSs and unstable $\uplambda _{max}$

**Table 2:** Mean core diameter $^{a}$ , hydrodynamic diameter and Zeta potential $^{b}$ of gold nanospheres in the absence and presence of alendronate, memantine, and tobramycin

Gold nanospheres	Core D. ± SD (nm)	Hydrodynamic D. ± SD (nm)	Zeta potential ± SD (mV)
AuNSs	9.58 ± 1.27	52.08 ± 3.54	− 24.56 ± 1.33
AuNSs with Alen (micro)	9.34 ± 1.07	58.94 ± 7.3	− 30.23 ± 3.61$^{c}$
AuNSs with Mema (nano)	–	64.99 ± 2.32	0.26 ± 0.25
AuNSs with Mema (micro)	9.59 ± 0.87	98.41 ± 1.05	0.53 ± 0.63
AuNSs with Tobr (nano)	10.01 ± 0.95	135.3 ± 4.75	− 23.63 ± 0.75
AuNSs with Tobr (micro)	9.90 ± 1.05	332.16 ± 38.54	− 1.64 ± 1.24

$^{a}$ The average core diameter was calculated from multiple HRTEM images

$^{b}$ The average hydrodynamic diameter and average Zeta Potential were calculated from three DLS metrics

$^{c}$ Unstable nanospheres, Zeta potential outside the range (− 30– +30 mV) are not stable

The molar concentration and extinction coefficient of gold nanosphere solutions in Table 1, have been calculated through the following steps:

1. Calculating the average number of gold atoms per nanosphere (N) from HRTEM images (Fig. 1). Assuming a spherical and fcc shaped nanosphere, the average number of gold atoms (N) per nanosphere was calculated using Eq. (1) [32], where $\pi$ is the circumference of a sphere (3.14), $\rho$ is the density for fcc gold ($19.3 g/cm^{3}$), $N_{A}$ is Avogadro’s number (the number of atoms per mole) ($6.02310^{23}$), M is the atomic weight of gold (197 g/mol), and D is the average core diameter of nanospheres that summarized in Table 2.
  1 $$\begin{aligned} N&= \frac{\pi \rho D^{3} N_{A}}{ 6M} =30.89602 D^{3} \end{aligned}$$
2. Calculating the molar concentration of the prepared solutions from initial concentration, using Eq. (2) where C is the molar concentration of the nanosphere solution, $N_{Total}$ is the total number of gold atoms (the initial amount of gold salt, $HAuCl_{4}$,used), N stands for the average number of gold atoms per nanosphere from Eq. (1), and V is the volume of the reaction solution in (L), assuming that the gold(III) reduction was complete.
  2 $$\begin{aligned} C&= \frac{N_{Total}}{NV} \end{aligned}$$
3. Determining the molar extinction coefficient of each sample from the slope of the regression line of absorbance vs concentration curves (Fig. 2). This actually based on Lambert-Beer’s Eq. (3), where A stands for absorbance, $\epsilon$ is molar absorptivity or molar extinction coefficient (the slope), b is the path length of cuvette (1cm), C is the calculated molar concentration of gold nanosphere solutions from Eq. (2).
  3 $$\begin{aligned} A = \epsilon bC \end{aligned}$$

### Gold nanospheres’ color stability, and UV-Vis absorbance spectra in the absence and presence of medications

**Fig. 3:**
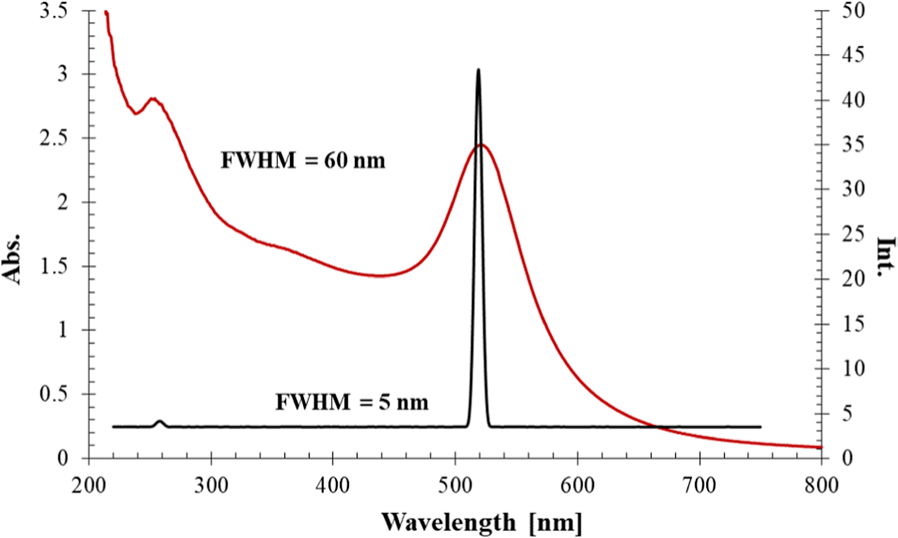
spectrophotomertic and spectrofluorimetric characterization of AuNSs at $\uplambda _{max}$ 520 nm & $\uplambda _{ex}$ 520 nm

The synthesized gold nanosphere solution ($3.6810^{-8}$ M) has been characterized using UV-Vis spectrophotometer with maximum absorption at $\uplambda _{max}$ 520 nm (Fig. 3).

**Fig. 4:**
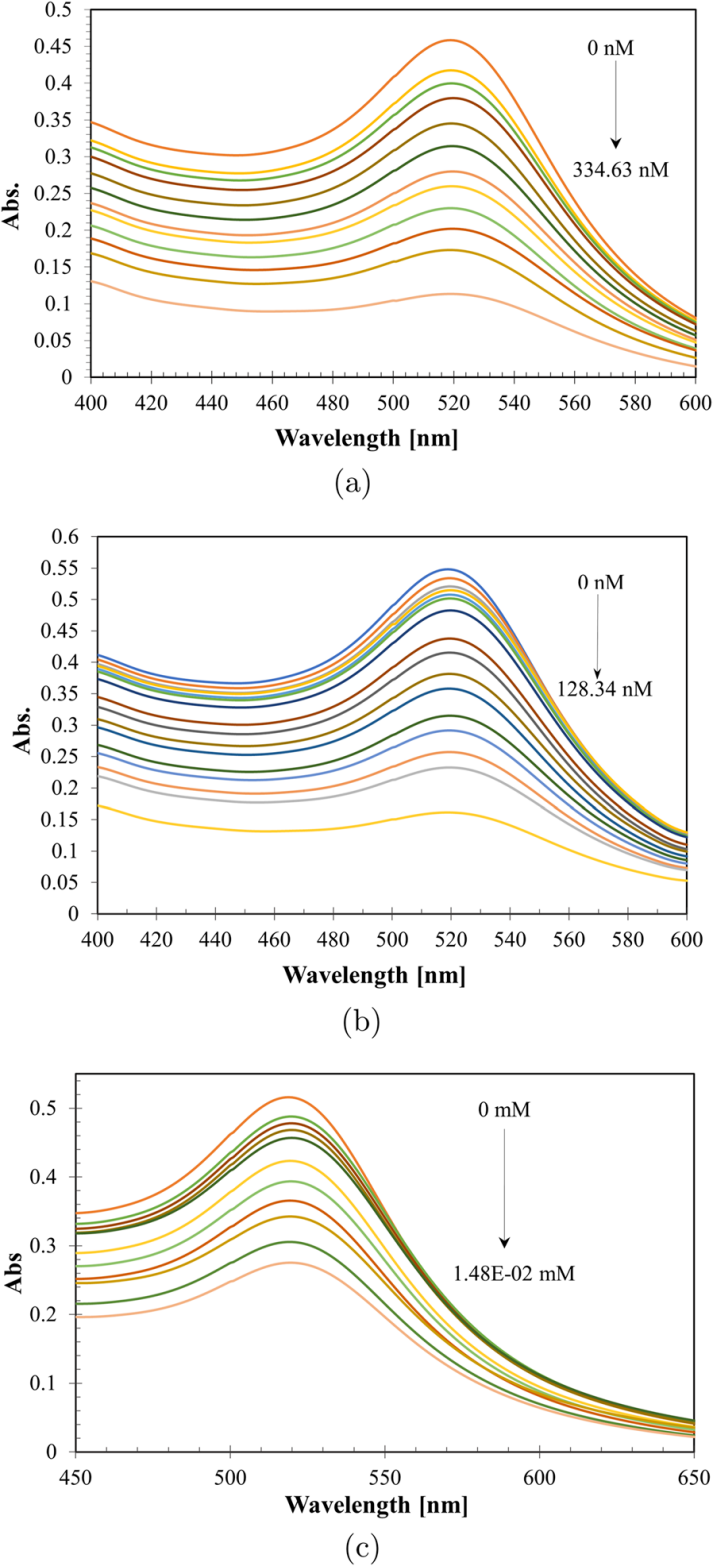
Absorbance spectra of gold nanospheres with Memantine nano-concentration within range of (33.46–334.63 nM) (**a**), Tobramycin nano-concentration within range of (4.28–128.34 nM) (**b**), Alendronate milli-concentration within range of (0.11–1.48 E–02 mM) (**c**)

The color stability of gold nanospheres (AuNSs) has been studied visually and by UV-Vis spectrophotometer. Visually, The color of AuNSs was stable for long period under tight storage conditions ($\simeq$ six months) [33]. After the addition of drugs to AuNSs, The color of AuNSs was mostly stable with alendronate, while least stable with memantine and tobramycin. The color change and the reactivity with tobramycin were most powerful than with memantine. Once the color has changed, the process continues until the color is finally gone. In the nano-scale, memantine and tobramycin made no color change. However, on the micro-scale, color change has occurred.

**Table 3:** PH changes of gold nanospheres’ solution in the absence and presence of medications

Gold nanospheres’ solution	$pH_{1 }$	$pH_{2}$	$pH_{3}$	Mean ± SD
AuNSs	5.89	5.45	5.34	5.56 ± 0.291
AuNSs with Alen (nano)	5.05	5.32	5.41	5.26 ± 0.187
AuNSs with Alen (micro)	5.02	4.99	5.1	5.03 ± 0.056
AuNSs with Mema (nano)	5.78	5.09	5.68	5.51 ± 0.372
AuNSs with Mema (micro)	5.94	6.08	5.98	6 ± 0.072
AuNSs with Tobr (nano)	5.35	5.45	5.71	5.50 ± 0.185
AuNSs with Tobr (micro)	6.09	6.61	6.11	6.27 ± 0.294
Mean pH of all AuNSs solutions				**(5.59 ± 0.118)**

**Table 4:** FT/IR important readings of gold nanospheres’ solution in the absence and presence of medications

Gold nanospheres’ solution	O–H and N–H stretching	C–H aliphatic stretching	C=O stretching	C–N and C–O stretching
AuNSs	3433.64	2917.77 and 2850.27	1698.98	1062.59
AuNSs with Alen (nano)	3439.42	2920.66	1631.48	1033.66
AuNSs with Alen (micro)	3434.6	2918.73	1628.59	1113.69
AuNSs with Mema (nano)	3439.71	2917.77 and 2851.24	1627.63	1056.8
AuNSs with Mema (micro)	3454.56	2919.7	1625.7	1056.8
AuNSs with Tobr (nano)	3441.35	2919.7	1630.52	1035.59
AuNSs with Tobr (micro)	3458.71	2922.59 and 2854.13	1632.45	1081.87

**Fig. 5:**
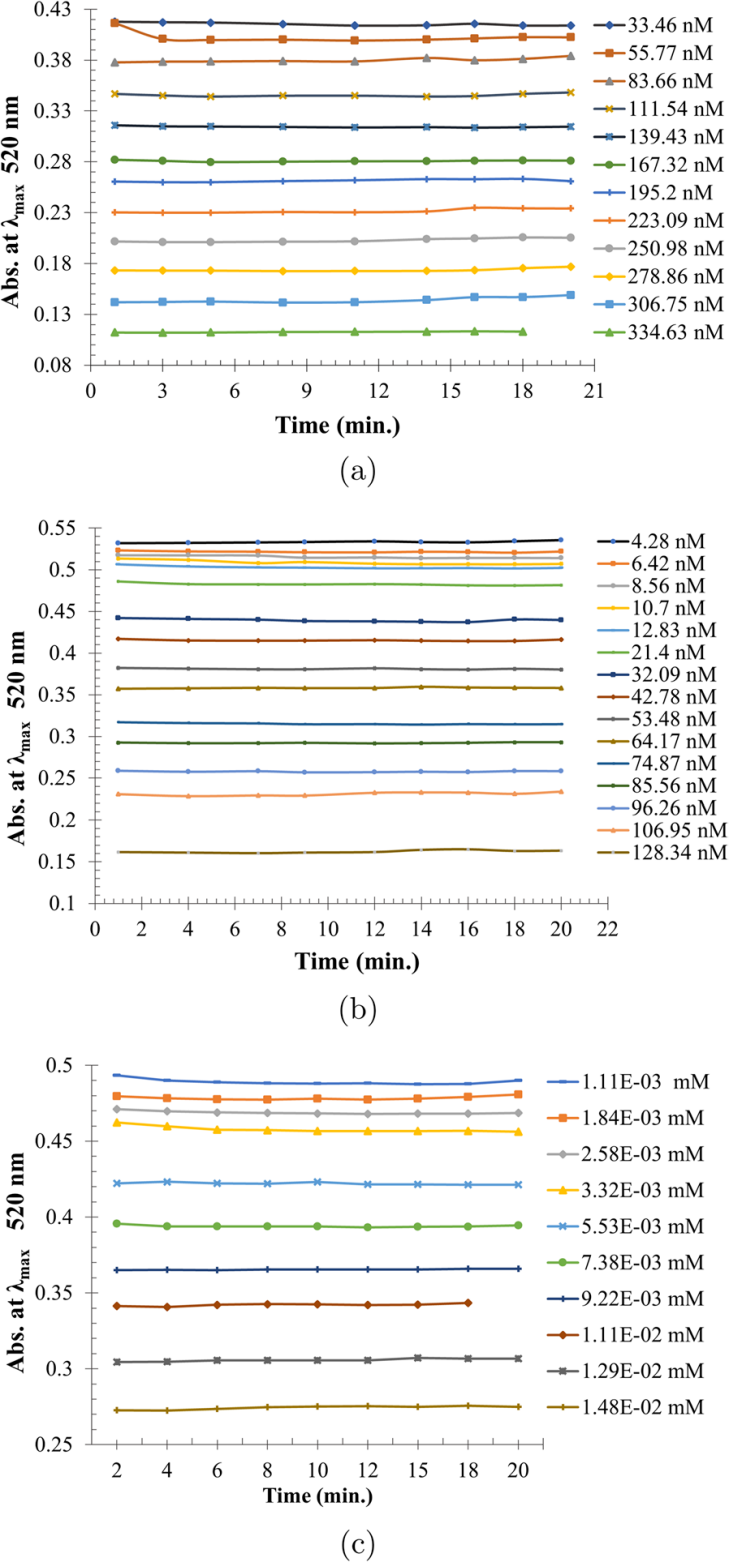
Twenty minutes stability of gold nanospheres with Memantine at nano-range (33.46–334.63 nM) (**a**), Tobramycin at nano-range (4.28–128.34 nM) (**b**), Alendronate at milli- range (0.11–1.48 E–02 mM) (**c**)

**Fig. 6:**
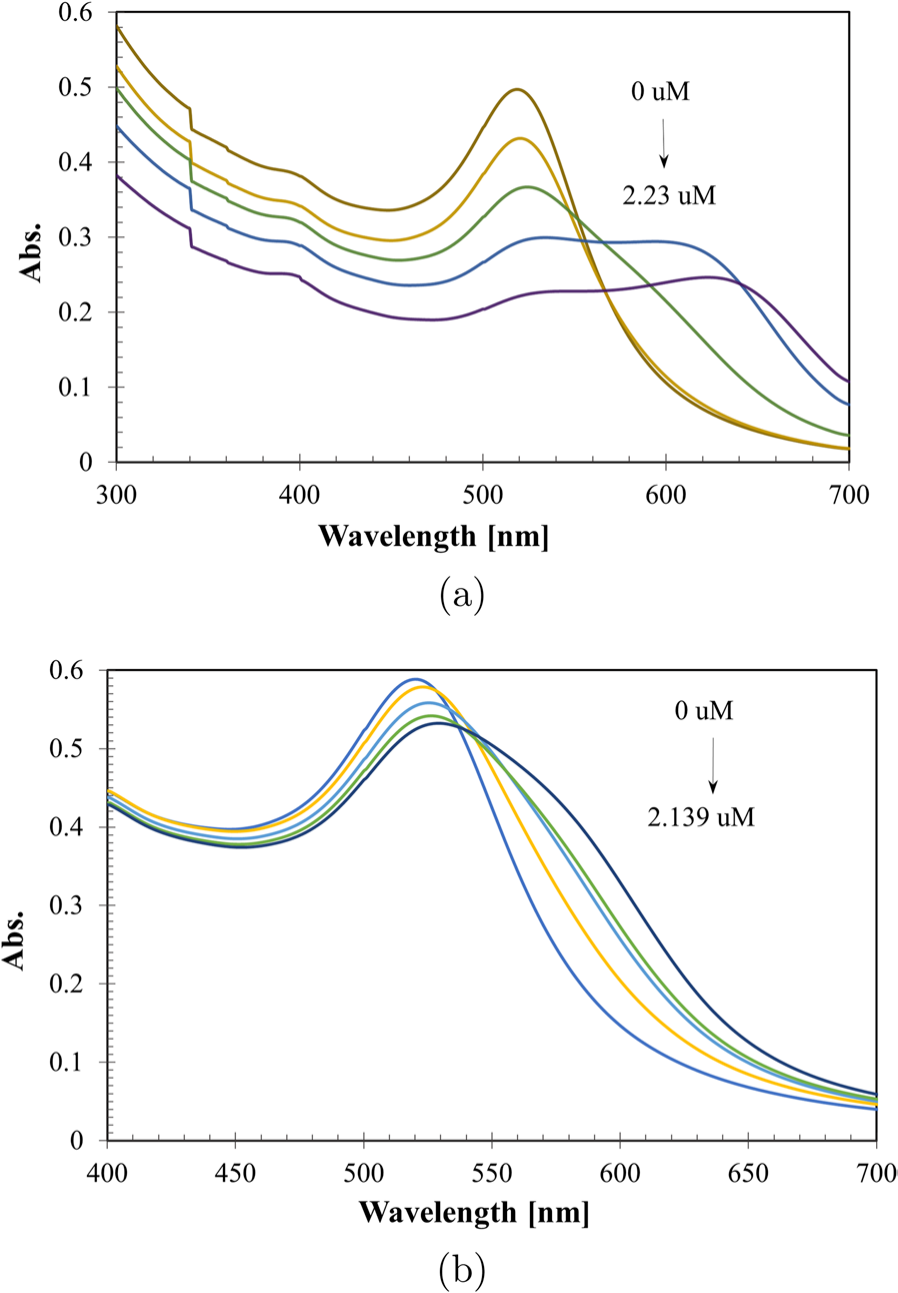
Redshifts in absorbance spectra of gold nanospheres loaded by memantine (micro-level) (**a**), and tobramycin (micro-level) (**b**)

**Fig. 7:**
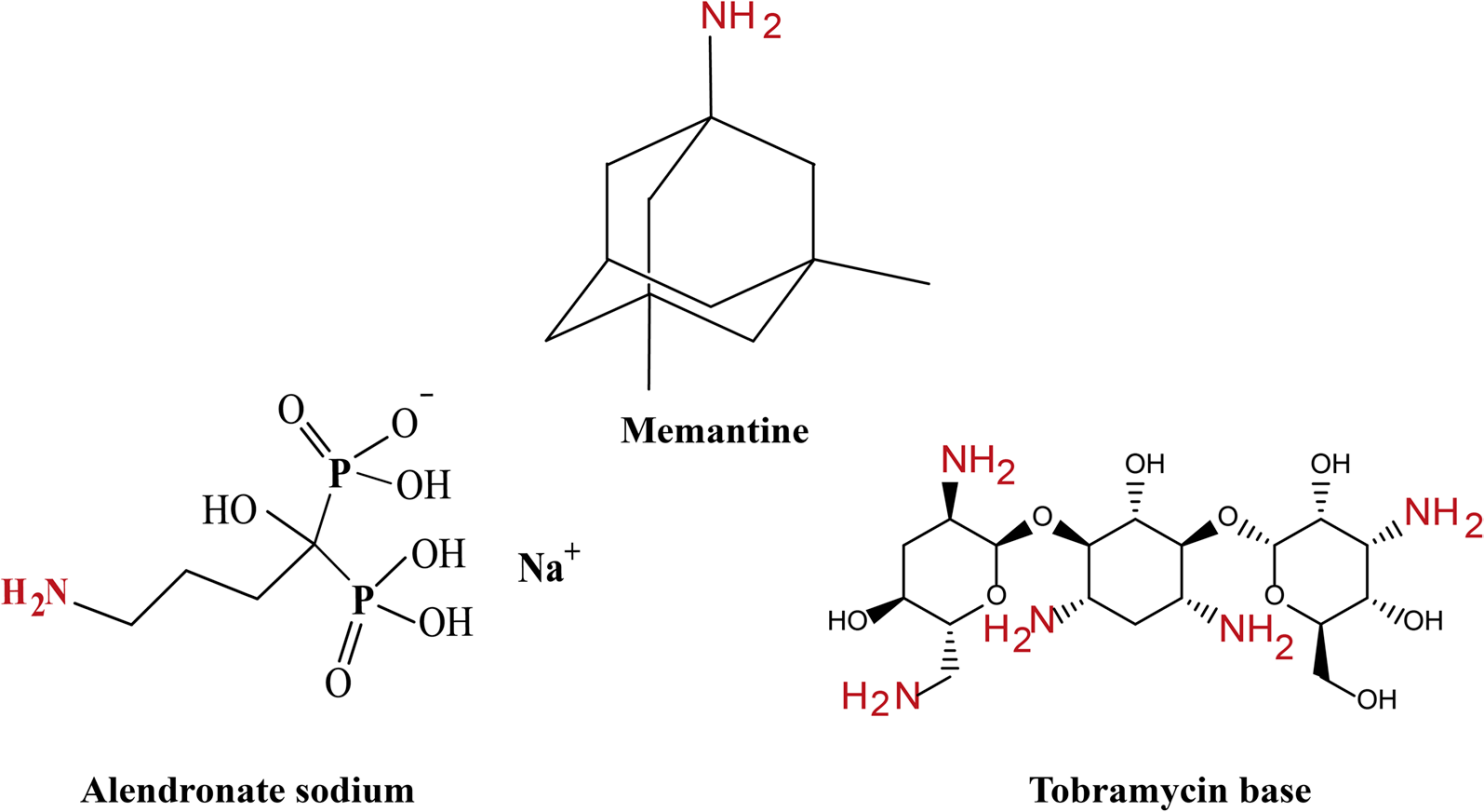
Chemical structures of alendronate, memantine, and tobramycin molecules

**Fig. 8:**
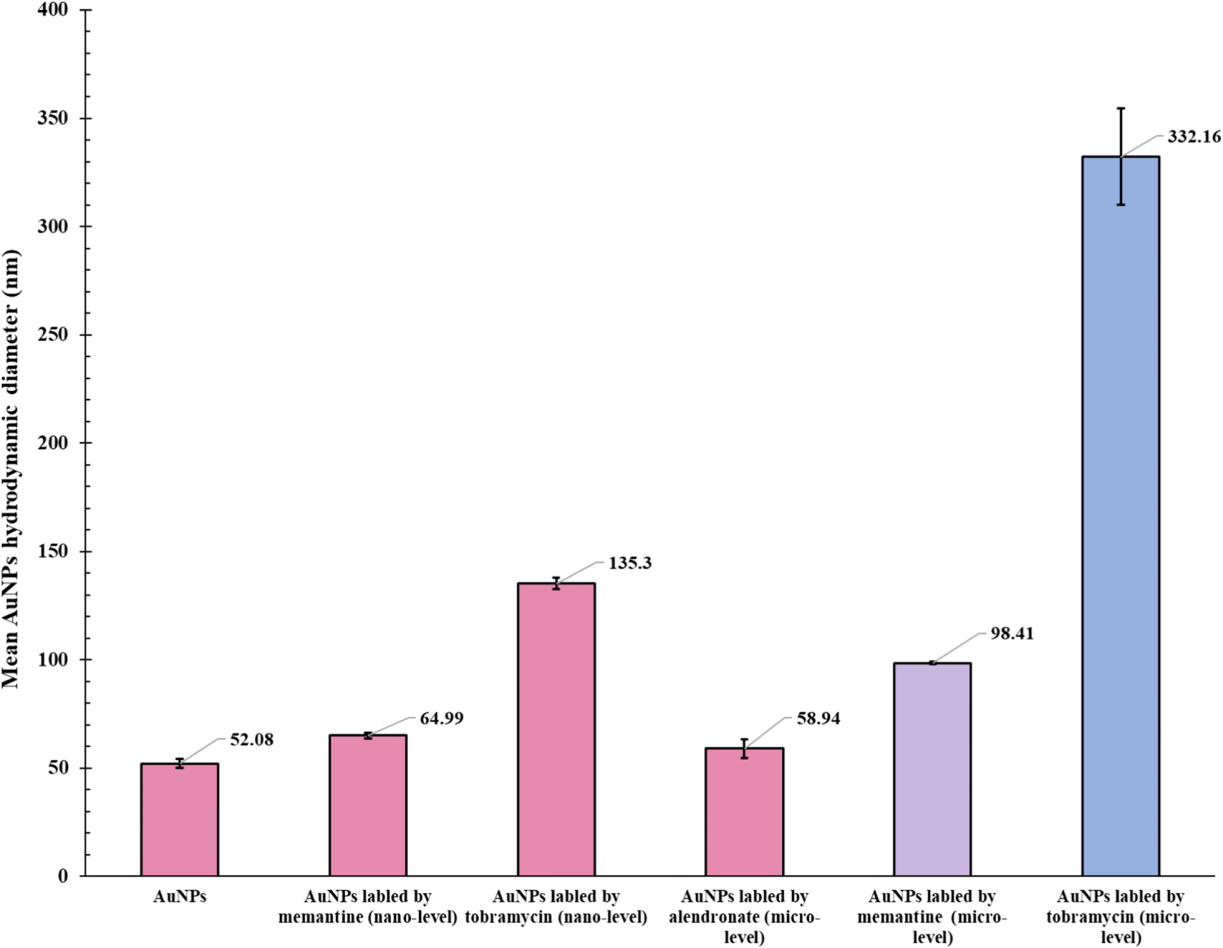
Average hydrodynamic diameter of gold nanospheres alone and with the various concentrations of alendronate, memantine, and tobramycin

**Fig. 9:**
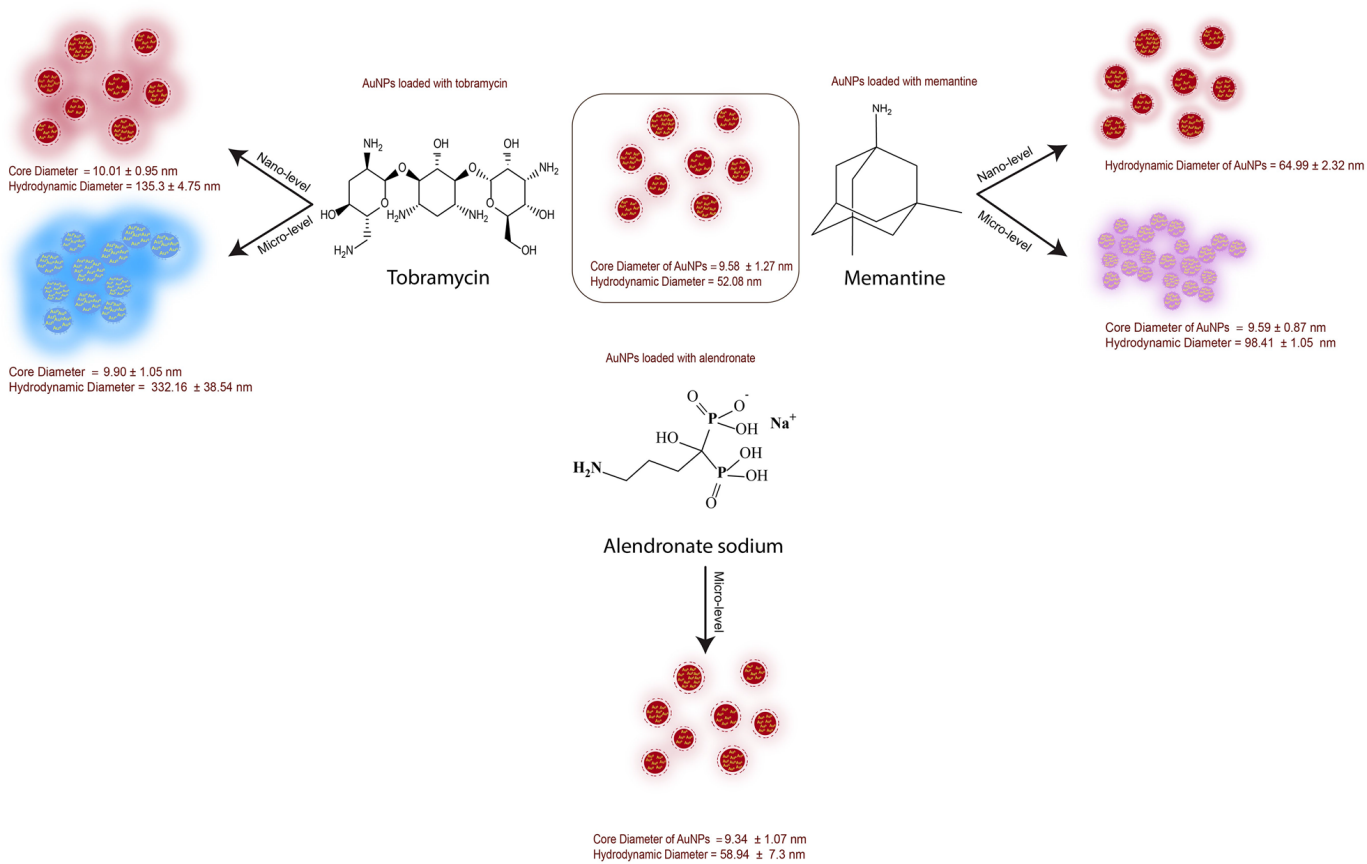
A graphical diagram shows how the colors change along with the hydrodynamic diameter and core diameter change

**Fig. 10:**
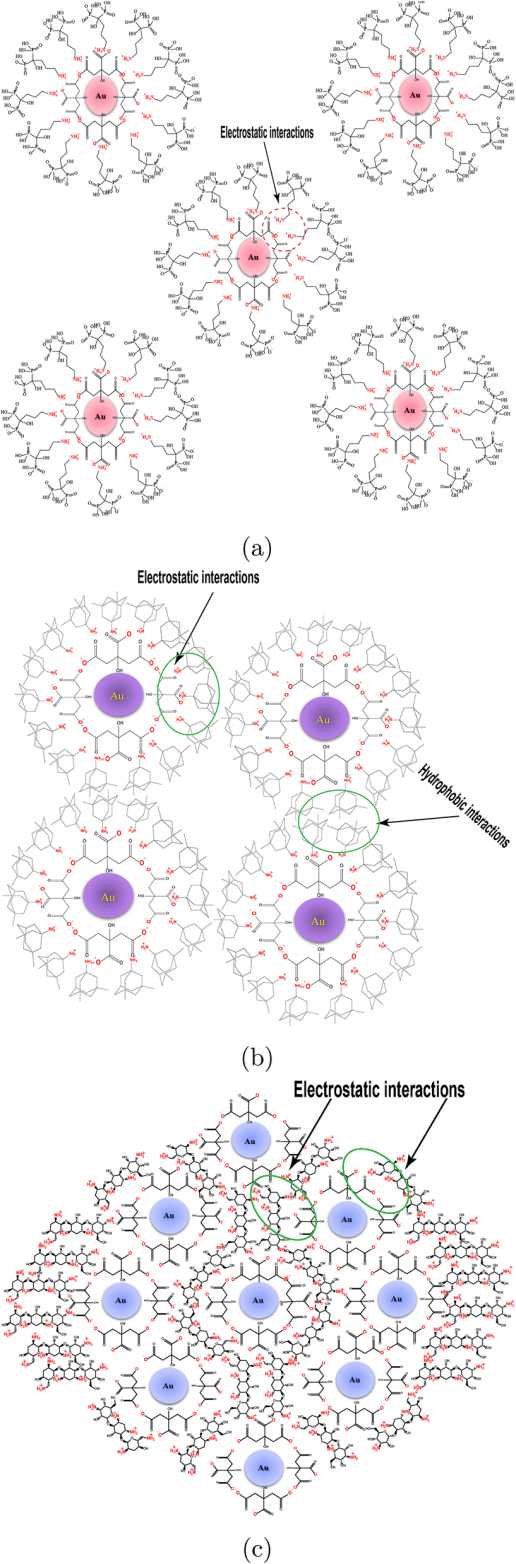
Illustrative diagram of proposed arrangement and aggregation of alendronate, memantine, and tobramycin toward gold nanospheres

**Fig. 11:**
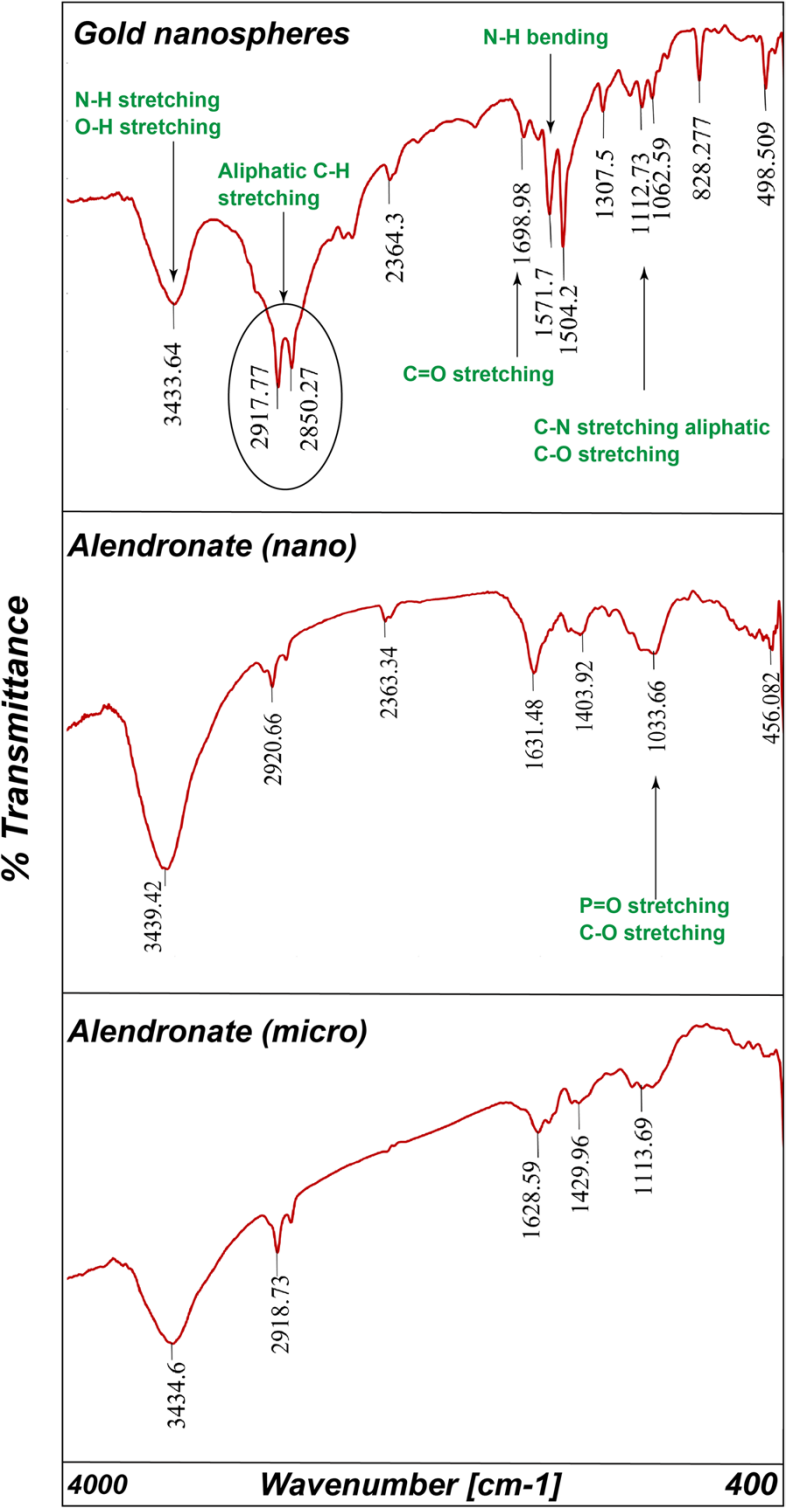
FTIR of gold nanospheres in absence and presence of alendronate

**Fig. 12:**
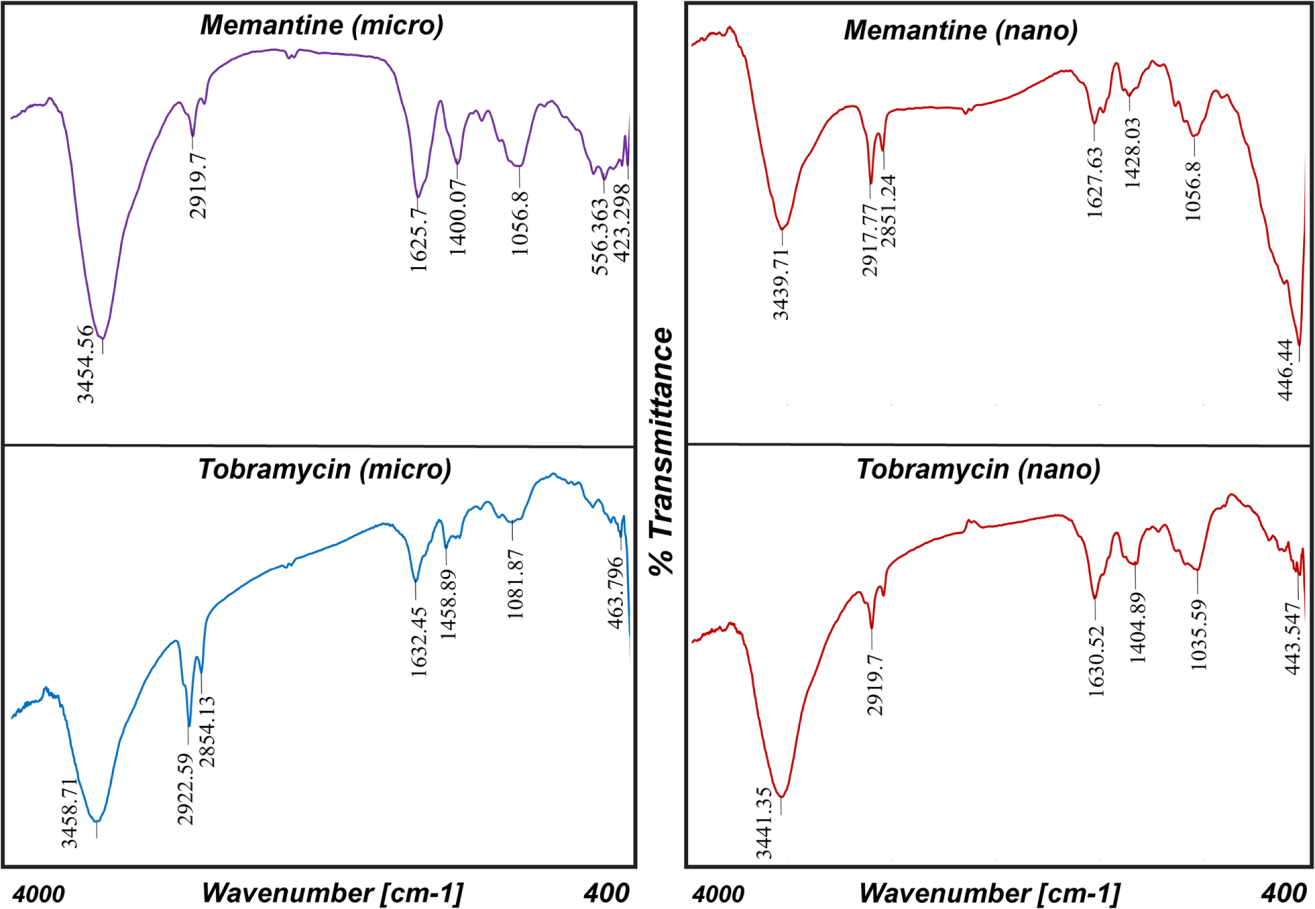
FTIR of gold nanospheres in presence of memantine and tobramycin

Using UV-Vis, the nano-ranges of memantine and tobramycin did not lead to redshift at $\uplambda _{max}$ 520 nm (Fig. 4a, b) or nanospheres’ aggregations (Fig. 1 ), also the milli-, micro-, or nano-ranges of Alen did not lead to redshift at $\uplambda _{max}$ 520 nm (Fig. 4c). Within 20 min, Mema nano-ranges (33.46–334.63 nM) and Tobr nano-ranges (4.28–128.34 nM) were almost stable (Fig. 5a, b), while Alen was stable in the milli-ranges (0.11–1.48 E–02 mM) (Fig. 5c). However, the micro-ranges of Mema & Tobr led to redshift (Fig. 6) and aggregation of gold nanospheres occurred (Fig. 1d, e).

### Gold nanospheres’ core diameter in the absence and presence of medications

HRTEM images indicated that the core diameters of gold nanospheres do not change when adding different medications of various concentrations even when nanospheres aggregated. The average core diameter of AuNSs was 9.58 ± 1.27 nm. After the addition of alendronate, memantine, and tobramycin to the AuNSs solution, the average core diameters were relatively stable (9.68 ± 0.15 nm) as obvious in HRTEM images (Fig. 1) and the calculated average core diameter in Table 2. The aggregation and color change of gold nanospheres occur due to factors other than an increase in the core size of gold nanospheres that could be due to an increase in hydrodynamic diameter of nanospheres, attraction and repulsion forces of the outermost layer of nanospheres, and the chemical compositions of drugs.

### Gold nanospheres’ hydrodynamic diameter, and surface charge in the absence and presence of medications

The average hydrodynamic diameter of gold nanospheres solution, calculated from DLS to be 52.08 ± 3.54 nm, increases with increasing drug concentrations, except with alendronate where the hydrodynamic diameter of particles showed a very small change of 58.94 ± 7.3 nm. In the case of tobramycin, the hydrodynamic diameter increases from 135.3 ± 4.75 nm to 332.16 ± 38.54 nm which is a large increase. While the hydrodynamic diameter in the case of memantine increases from 64.99 ± 2.32 nm to 98.41 ± 1.05 nm. This may be attributed to the bulk structure of tobramycin and its various functional groups as in Fig. 7 which accelerate the accumulation of more drug molecules on the surface of gold nanospheres. While the memantine structure is very small and has only one primary amine functional group available for reaction. The average hydrodynamic diameters of gold nanospheres ’ solutions have been summarized in Table 2 and illustrated in Fig. 8.

A graphical illustration of changes in the AuNSs core and hydrodynamic diameter showed in Fig. 9.

Similarly, zeta potentials were measured in triplicate to analyze the surface charge of gold nanospheres. Gold nanospheres’ solutions’ average zeta potentials were summarized in Table 2, the greatest decrease in negativity was with memantine (from 0.26 to 0.53 mv), while a decreased negativity with tobramycin (from − 23.63 to − 1.64 mv) in the micro-level. But with alendronate, a slight increase in negativity (from − 24.56 to − 30.23 mv) occurred.

### Gold nanospheres solutions’ pH changes in the absence and presence of medications

To better understand how these medications interact with gold nanospheres, it was crucial to look into the pH changes in these experiments. The pH of gold nanosphere solutions remained within an acidic range (5.03–6.27) as shown in Table 3. The micro-concentration of alendronate sodium produced the highest acidity (pH= 5.03), whereas the micro-concentration of tobramycin produced the lowest acidity (pH= 6.27). These results may support the interaction and arrangement of medications around the gold nanospheres as illustrated in Fig. 10, where the majority of the primary amine groups ($-NH_{2}$) will be protonated to ($-NH_{3} ^{+}$) in the acidic media and thus interact with the carboxylic acid groups ($-COO^{-}$) of citrate-anions.

### Gold nanospheres’ FTIR spectra in the absence and presence of medications

Further to understand how these medications interact and maintain their stability with gold nanospheres, FTIR measurements have been performed. FTIR spectra of AuNSs showed a strong, broad, and stretching vibrational peak at 3433.64 $cm^{-1}$ which corresponds to O–H and N–H stretching of aliphatic primary amine. These broad peaks indicate electrostatic interactions (hydrogen bonding or protonation of primary amine) as it shifts to higher wavenumbers (Table 4) with all medications. There is a sharp vibrational peak of C–H aliphatic stretching at 2917.77 $cm^{-1}$ and 2850.27 $cm^{-1}$ which shifts to higher wavenumbers with all medications at all concentration levels except with memantine at nano-concentration remained constant (Fig. 12). This shift indicates hydrophobic interactions. The stretching vibrational peak at 1698.98 $cm^{-1}$ of C=O of the carboxylic group shifts to lower wavenumbers which indicates consumption of carboxylate anion in the electrostatic interactions. An aliphatic stretching of C–N and C–O peaks at 1112.73 $cm^{-1}$ and 1062.59 $cm^{-1}$ which shift to lower wavenumbers with all medications (Table 4). The characteristic peak for P=O of alendronate (Fig. 11) is very diminished and broad which indicates its consumption in electrostatic interactions.

### Proposed gold nanospheres’ reactivity mechanism

The stabilization and reactivity of Turkevich gold nanospheres are attributed to various mechanisms including but not limited to the electrostatic attractive repulsive interactions of the negative coating layers of citrate-anions with the nanosphere core which keep the particles suspended in the colloidal solution without precipitation [32]. The suggested stability of Turkevich gold nanospheres after the addition of solutions of the tested drugs (Alen, Mema, Tobr) could be explained from their chemical structures (Fig. 7) and the above-mentioned results as follows. The acidic environment which was preserved with all medications at all concentration levels (pH= 5.03–6.27), will lead to protonation of the primary amine group ($-NH_{2}$) which is present in the three medications. These protonated primary amine groups ($-NH_{3}^{+ }$) suppose to interact with the negative charge of carboxylic acid groups ($-COO^{-}$) of citrate-anions through the electrostatic attractive mechanism. In the case of alendronate, there is only one primary amine group ($-NH_{2}$) that would interact with the weak carboxylic acid groups ($-COO^{-}$) of citrate-anions, leaving the bis-phosphonate group ($-COH(PO_{3}H_{2} )_{2}$) freely coating the outermost layer of nanospheres. This second negative layer of the bis-phosphonate group could be another stabilizing, and protective layer, which may contribute to the compatibility of gold nanospheres with alendronate through a repulsive mechanism (Fig. 10a).

On the other hand, in spite of memantine also contains only one primary amine group ($-NH_{2}$) like alendronate, that suppose to be protonated and interact with the weak carboxylic acid group ($-COO^{-}$) till complete saturation of the outermost layer of gold nanospheres with memantine. But on the other side of the molecule, there are uncharged hydrocarbon chains that are completely different from the negative charge bis-phosphonate group. It was observed that memantine exhibits positive zeta potential as in Table 2 which could be explained by the presence of the hydrocarbon chains, that may form hydrophobic interactions with each other, leading to the attraction and aggregation of gold nanospheres to each other at the micro-level (Fig. 10b).

On the contrary, Tobramycin which contains five primary amine groups ($-NH_{2}$), will interact faster with the gold nanospheres and in many directions. Tobramycin will interact through an electrostatic attractive mechanism (Fig. 10c). This may explain why tobramycin has rapid reactivity toward these nanospheres.

There is also another possible mechanism of aggregation which may be attributed the stereochemistry of the three medications and the way of arrangement of these medications around gold nanospheres during the interaction.

For reactivity, it was obvious that the reactivity of nanospheres with medications depends on both the structure bulkiness and the concentrations used. Therefore, the reactivity order could be arranged as follows: tobramycin (micro-level) > memantine (micro-level) > alendronate (milli-level).

One of the explanations for the nanocrystals’ reactivity, color change, and aggregation could be the increase in hydrodynamic diameter and the decrease in zeta potential (negativity). Both of these parameters already change in the case of memantine and tobramycin while remaining almost stable with alendronate. All reaction mechanisms have been graphically illustrated in Figs. 9 and 10.

## Conclusion

Utilizing hydrophilic gold nanospheres (AuNSs), this study examined the effects of three formulations: alendronate, memantine, and tobramycin on the morophostructural features and stability of AuNSs. These medications were included as free water-soluble, non-chromophoric, and targeted medications. The optimum conditions for reactivity and compatibility were studied through UV-Vis, HRTEM, Zeta potential, particle size, pH changes, and FTIR measures. Based on that, AuNSs were mostly stable with alendronate sodium in all concentration levels (milli-, micro-, and nano-), while memantine and tobramycin showed stability in the nano-concentration only (no aggregation and color change). The AuNSs core diameter was almost constant within a range of $9.34 -10.01 \, nm$, and the molar concentration remained constant within a range of $3.22 - 3.97 * 10^{-8} M$ with all medications, while the hydrodynamic diameter increased with increasing drug concentration except with alendronate, a negligible increase occurred. Conversely, Zeta potential decreased as concentrations increased. The pH remained acidic within the range of 5.03– 6.27, and this explains how these medications could interact with nanospheres either through hydrophobic or electrostatic interactions. FT/IR spectra showed electrostatic interactions through the protonation of primary aliphatic amines and hydroxylic groups, consumption of carboxylate anions, and phosphate groups. While hydrophobic interactions occurred through the consumption of aliphatic C–H groups. Finally, to avoid aggregation of gold nanospheres for the purpose of targeted drug delivery in reactive medications such as memantine and tobramycin, concentrations should be lowered to as nano-levels as possible.

## Data Availability

All data generated or analysed during this study are included in this published article.
